# A dataset of US precinct votes allocated to Census geographies with precision

**DOI:** 10.1038/s41597-025-05140-3

**Published:** 2025-05-15

**Authors:** Amir Fekrazad

**Affiliations:** College of Business, Texas A&M University-San Antonio, 1 University Way, San Antonio, Texas 78224 USA

**Keywords:** Politics, Interdisciplinary studies

## Abstract

Voting precincts are the finest spatial units for recording U.S. election results, while census geographies, including block groups, census tracts, and ZIP Code Tabulation Areas (ZCTAs), provide administrative data on demographic, economic, health, and environmental factors. This paper presents datasets that link precinct-level voting records to census geographies with precision. The allocation assumes votes within a precinct are proportional to household population, with population distributed from block groups to overlapping precinct fractions using the Regional Land Cover Regression (RLCR) method. Datasets based on surface area and imperviousness methods are also provided, but RLCR outperforms them across multiple error metrics in validation tests using census blocks and voter-level data. Covering the 2016 and 2020 U.S. general elections, these datasets facilitate merging voting records with sources like the American Community Survey, CDC Places, and IRS Statistics of Income to explore voter behavior across various contexts.

## Background & Summary

Precincts are the smallest geographic units for tabulating votes in U.S. elections, but government agencies do not provide non-electoral data, such as demographic and socioeconomic information, at this level. In contrast, census geographies, such as census tracts and ZCTAs, are widely used for reporting such data. Linking precinct votes to these geographies opens opportunities for detailed analyses of voting behavior in relation to demographic, economic, health, and environmental factors. To support such integration, this paper presents datasets developed through a reproducible and rigorously defined framework for mapping precinct-level voting records onto census geographies.

Research studies that involve U.S. election results, whether as the outcome being analyzed or as an explanatory factor for other phenomena, typically rely on data aggregated at the county level, as seen in works such as^1–5^, and^6^. Another commonly used resource for nationwide studies is the American National Election Studies (ANES) (e.g.^7–9^), which also lacks granular geographic detail. This limitation can obscure important variations in voting patterns.

Consider a county that contains both predominantly blue-collar and predominantly white-collar areas. Using county-level data, the county appears as a homogenous mix of these groups (e.g., 50% blue-collar, 50% white-collar) and their aggregated voting behavior. This masks the distinct voting patterns of each group. In contrast, finer geographic resolutions, such as census tracts, preserve these variations and enable researchers to better identify their impact on electoral outcomes.

The datasets were created based on the assumption that, within a precinct, votes are proportional to household population. The process begins by intersecting precinct boundaries with block groups, the smallest census units with annual population estimates. This intersection divides precincts and block groups into smaller fractions, each uniquely assigned to a single precinct and a single block group. Block group populations are distributed among their fractions according to land cover classes (e.g., low-, medium-, or high-intensity development, farmland, forest). Precinct votes are then allocated to the fractions based on these population estimates and aggregated at the block group, census tract, and ZCTA levels.

Dasymetric mapping using land cover has been explored in various forms, as seen in^10,11^, and^12^. The approach underlying this paper’s datasets, Regionalized Land Cover Regressions (RLCR), is distinct in that block groups are first clustered to form the smallest possible source zones. Within each cluster, a constrained regression model estimates the population contribution of each land cover category. This model is then applied to predict the population of fractions within the same cluster.

This study also provides datasets allocating votes to census geographies using population distributions based on surface area and non-road imperviousness^13^ methods. Validation tests allocating 2020 block group populations to census blocks against actual block populations show that RLCR achieves higher predictive accuracy than alternative methods. A separate test comparing North Carolina individual voter counts aggregated to census tracts with vote counts generated by all methods also demonstrates RLCR’s superior accuracy.

The datasets can be easily integrated with a wide array of administrative data to explore different aspects of voting behavior. For example, merging with ACS data^14^ makes it possible to analyze the impact of demographic, socioeconomic, housing, and labor factors. Merging with IRS ZIP code-level Statistics of Income (SOI)^15^—noting that ZCTAs and ZIP codes do not align perfectly but ZCTAs are a sufficient approximation—allows for examining interactions between voting patterns and tax behavior. Integrating with local election laws and regulations provides a large sample with substantial statistical power to investigate the disparate impact of these laws on disadvantaged groups.

Additionally, combining the datasets with CDC Places tract-level health data^16^ allows researchers to examine how public health issues influence voting. Similarly, linking with FEMA tract-level data^17^ on the Risk Index, Expected Annual Loss, Social Vulnerability, and Community Resilience can offer insights into how climate and environmental factors shape voting patterns and party preferences.

To showcase the importance of precision in allocating precinct votes to census geographies and its impact on research outcomes, a simple voter turnout model was developed using a small set of demographic and socioeconomic variables. The findings reveal that some coefficients estimated using the naive areal method differ significantly from those derived using RLCR, particularly in rural regions where larger geographic areas and uneven population densities pose challenges for accurate vote allocation.

## Methods

This section introduces the data sources used and outlines the process of synthesizing them to create the vote allocation datasets.

### Inputs

The primary data sources used to create the datasets are described below. These sources are all publicly available without fees or membership requirements, provided by government or research entities under public domain or open licenses.

#### Voting Precincts

Precincts are the main units for assigning residents to polling places and distributing ballots customized for local races and issues. Each precinct records the votes of residents within its boundaries, including those cast by mail, and reports the results to higher election authorities.

This study used precinct boundaries and U.S. general election results provided by^18^ for 2016 and^19^ for 2020, with the methodologies detailed in^20^ and^21^, respectively. While presidential election results are available for all states, results for other races may vary in coverage across states.

In 2016 and 2020, the conterminous United States contained about 176,500 and 177,600 voting precincts, respectively. On average, each precinct recorded approximately 800 votes in 2016 and 900 votes in 2020.

#### Census Geographies

Census geographies are administrative boundaries established by the U.S. Census Bureau to organize and report population and socioeconomic data. These boundaries are typically updated every 10 years with new decennial census data to reflect population changes, though minor adjustments may occur between censuses.

Census blocks represent the smallest geographic unit, corresponding to urban blocks or small rural areas. Block groups aggregate multiple census blocks and serve as a higher-level unit for statistical analysis. Census tracts consist of 1 to 9 block groups and are commonly used for detailed statistical reporting. Finally, ZCTAs are approximations of postal ZIP codes, constructed from census blocks.

All these geographic boundaries are publicly accessible through the Census TIGER/Line Shapefiles; for this study, those from 2016^22^ and 2020^23^ were used.

Although census blocks provide the finest resolution, their data are only available during decennial census years. Since elections often do not coincide with these years, block groups were used as the smallest census geography to ensure methodological consistency across different elections. Annual total and household population estimates are available for block groups through ACS^24,25^ (tables B01003 and B11002, respectively).

#### Land Cover Raster Maps

In this study, land cover data from the National Land Cover Database (NLCD) for 2016 and 2020^26^ served as the main inputs for modeling population distributions within precincts. The NLCD assigns each 30 × 30-meter pixel across the conterminous United States to one of several land cover categories, including open water, low-, medium-, and high-intensity developed areas, farms, forests, and more.

Land cover evolves over time due to human development and climatic changes. Therefore, it is important to use land cover data that corresponds to the year of other data sources (e.g., the 2020 land cover raster for the 2020 elections). The high spatial resolution and detailed classification of land cover types make NLCD data particularly well-suited for refining population distribution estimates.

A downside of using land cover maps as an input in the process is that these maps are not routinely available for Alaska and Hawaii, limiting the applicability of the method to the conterminous United States.

All GIS processes projected vector and raster layers to the Albers Equal Area (WGS84) coordinate system for consistency and accuracy.

### Allocation Process

The allocation of precinct votes to census geographies was based on the assumption that voting outcomes, both turnout and candidate tallies, are proportionally distributed within the precinct based on household population. Formally, consider a precinct with a total of *T* votes, of which *c* are for candidate *C*. If the precinct is divided into fractions (subregions), and one fraction contains *r*% of the precinct’s household population, that fraction is assigned *r* ⋅ *T*/100 total votes and *r* ⋅ *c*/100 votes for candidate *C*.

Precincts are already the most granular level at which vote counts are publicly reported, and for privacy reasons, no data exists that reveals individual-level vote choices. As a result, the assumption that voting outcomes are proportionally distributed across subregions of a precinct cannot be tested. Nevertheless, because precincts are typically small and tend to encompass relatively homogeneous populations in terms of socioeconomic and demographic characteristics, this assumption remains plausible.

Preliminary analysis revealed that in every state, a small number of block groups or census tracts have a high concentration of correctional facilities. These areas report large populations but should receive fewer votes, as most residents are ineligible to vote. To address this, votes were distributed based on household population rather than total population. The household population excludes individuals living in group quarters, both institutionalized (e.g., prisons) and non-institutionalized (e.g., nursing homes). This adjustment improved the precision of the results.

Building on the described data inputs and assumptions, the following procedure was used to allocate precinct vote records to block groups, census tracts, and ZCTAs.

#### Step 1: Intersecting Precincts and Block Groups

The first step involves intersecting precinct boundaries with block groups, the smallest geographic units for which annual population estimates are available. Since census tracts are composed of block groups, there is no need to further intersect them with precincts. However, for ZCTAs, an additional intersection with ZCTAs is required because they do not fully align with block groups.

This process divides each precinct into smaller areas, called fractions, each fully contained within a single block group (and a single ZCTA, if applicable).

To exclude intersections that merely touch at their boundaries or have negligible overlap, only fractions with surface areas exceeding 0.1% of either intersecting feature are retained.

**Fig. 1:**
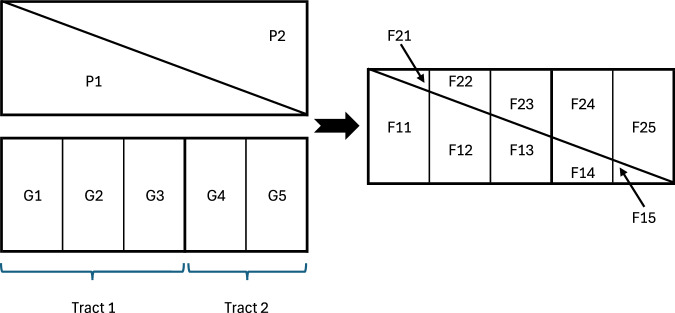
An illustration of precinct and census geography intersection.

To illustrate this process, Fig. 1 depicts an area containing two precincts (*P*_1_ and *P*_2_) and two census tracts. One tract consists of three block groups (*G*_1_, *G*_2_, *G*_3_), while the other contains two block groups (*G*_4_ and *G*_5_). Vote counts are known for precincts, and population counts are known for block groups.

Intersecting the precincts and block groups produces 10 fractions, indexed as *F*_*pg*_, where *p* denotes the precinct number and *g* the block group number.

#### Step 2: Distributing Population to Fractions

The household population of a block group *G*_*g*_ is distributed to fractions *F*_*pg*_ within it using weights (*w*_*pg*_). These weights, which represent population density within the block group, will be discussed later. The population of the fraction is calculated as follows:

1 $$\,{\rm{Pop}}\,({F}_{pg})=\left(\frac{{w}_{pg}}{{\sum }_{{p}^{{\prime} }:{F}_{{p}^{{\prime} }g}\in {G}_{g}}{w}_{{p}^{{\prime} }g}}\right)\times \,{\rm{Pop}}\,({G}_{g})$$

where the denominator sums the weights of all fractions within block group *G*_*g*_.

In the figure’s example, the populations of *F*_13_ and *F*_23_ are calculated as follows:

$$\begin{array}{l}\,{\rm{Pop}}({F}_{13})=\frac{{w}_{13}}{{w}_{13}+{w}_{23}}\times {\rm{Pop}}\,({G}_{3})\\ \,{\rm{Pop}}({F}_{23})=\frac{{w}_{23}}{{w}_{13}+{w}_{23}}\times {\rm{Pop}}\,({G}_{3})\end{array}$$

#### Step 3: Allocating Votes to Fractions

Once populations are assigned to the fractions, precinct *P*_*p*_ votes are allocated to the fractions in proportion to their population shares within the precinct:

2 $$\,{\rm{Votes}}({F}_{pg})=\frac{{\rm{Pop}}({F}_{pg})}{{\sum }_{g{\prime} :{F}_{pg{\prime} }\in {P}_{p}}{\rm{Pop}}({F}_{pg{\prime} })}\times {\rm{Votes}}\,({P}_{p})$$

where the denominator represents the sum of the populations of all fractions within precinct *P*_*p*_.

In the example shown, the votes for fraction *F*_13_ are calculated as follows:

$$\,{\rm{Votes}}({F}_{13})=\frac{{\rm{Pop}}({F}_{13})}{{\rm{Pop}}({F}_{11})+{\rm{Pop}}({F}_{12})+{\rm{Pop}}({F}_{13})+{\rm{Pop}}({F}_{14})+{\rm{Pop}}({F}_{15})}\times {\rm{Votes}}\,({P}_{1})$$

#### Step 4: Aggregating Votes to Census Geographies

Finally, the total votes for a block group, tract, or ZCTA are calculated by summing the votes of all fractions within the respective geography.

3 $$\,{\rm{Votes}}({{\rm{CensusUnit}}}_{u})=\sum _{p{\prime} ,g{\prime} :{F}_{p{\prime} g{\prime} }\in {{\rm{CensusUnit}}}_{u}}{\rm{Votes}}\,({F}_{p{\prime} g{\prime} })$$

In the described example, the votes for block groups *G*_1_, *G*_2_, and *G*_3_ are calculated as:

$$\begin{array}{l}\,{\rm{Votes}}({G}_{1})={\rm{Votes}}({F}_{11})+{\rm{Votes}}\,({F}_{21})\\ \,{\rm{Votes}}({G}_{2})={\rm{Votes}}({F}_{12})+{\rm{Votes}}\,({F}_{22})\\ \,{\rm{Votes}}({G}_{3})={\rm{Votes}}({F}_{13})+{\rm{Votes}}\,({F}_{23})\end{array}$$

The votes for Tract_1_ are then calculated as:

$$\,{\rm{Votes}}({{\rm{Tract}}}_{1})={\rm{Votes}}({G}_{1})+{\rm{Votes}}({G}_{2})+{\rm{Votes}}\,({G}_{3})$$

### Determining Weights for Population Distribution

The precision of this process hinges on accurately determining the weights (*w*_*pg*_) for population distribution in Equation (1). Three potential methods for calculating these weights were explored.

#### Method 1: Areal

The simplest approach is to set *w*_*pg*_ proportional to the surface area of the fraction. It assumes a uniform population distribution within block groups.

$${w}_{pg}=\,{\rm{Surface\; Area}}\,({F}_{pg})$$

While straightforward, this assumption is often unrealistic, as population density can vary significantly within a block group.

#### Method 2: Imperviousness

A more sophisticated approach is derived from^13^, which utilizes surface impervious percentages and descriptors from the NLCD for 2016 and 2020^27,28^. Impervious percentage (ImpPct) is represented as a number (0-100) in raster data, where each pixel covers 30 × 30 meters. Additionally, the impervious descriptor identifies whether a pixel is a road or not.

The underlying assumption is that higher impervious percentages for non-water body pixels (ImpPct > 1) and non-road pixels indicate a greater likelihood of development and habitability. Thus, the habitability of a pixel is calculated as:

4 $${{\rm{Habitability}}}_{{\rm{pixel}}}={{\rm{ImpPct}}}_{{\rm{pixel}}}\times 1({{\rm{ImpPct}}}_{{\rm{pixel}}} > 1)\times 1({{\rm{NotRoad}}}_{{\rm{pixel}}})$$

where 1(⋅) is an indicator function that takes the value 1 if the condition inside is satisfied and 0 otherwise.

The habitability of an area is then defined as the sum of the habitability values for all pixels within it. This was used as the weight for distributing population among fractions in Equation (1):

5 $${w}_{pg}=\,{\rm{Habitability}}\,({F}_{pg})=\sum _{{\rm{pixel}}\in {F}_{pg}}{{\rm{Habitability}}}_{{\rm{pixel}}}$$

#### Method 3: Regionalized Land Cover Regression (RLCR)

This study’s main method leverages land cover data and a constrained regression model to estimate population contributions from various land cover categories (e.g., high-, medium-, and low-intensity developments, farmlands, and forests) within the smallest possible geographic regions. The model is then used to predict the population of sub-regions based on their respective land cover characteristics.

First, block groups and fractions are overlaid on the land cover raster, and the number of pixels for each land cover category is counted within each block group and fraction. These counts are the foundation of this method.

Next, the relationship between population and land cover categories for a block group *G* is described by the following equation:

6 $$\begin{array}{rcl}\,{\rm{Pop}}\,(G) & = & {b}_{21}\cdot {n}_{21}(G)+{b}_{22}\cdot {n}_{22}(G)+{b}_{23}\cdot {n}_{23}(G)+{b}_{24}\cdot {n}_{24}(G)\\  &  & +\,{b}_{31}\cdot {n}_{31}(G)+{b}_{41}\cdot {n}_{41}(G)+{b}_{42}\cdot {n}_{42}(G)+{b}_{43}\cdot {n}_{43}(G)\\  &  & +\,{b}_{52}\cdot {n}_{52}(G)+{b}_{71}\cdot {n}_{71}(G)+{b}_{81}\cdot {n}_{81}(G)+{b}_{82}\cdot {n}_{82}(G)+{\epsilon }_{G},\\ {b}_{i} & \ge  & 0.\end{array}$$

where *n*_*i*_(*G*) represents the number of pixels in land cover category *i* within block group *G*, *b*_*i*_ is the coefficient that quantifies the population contribution of that category, and *ϵ*_*G*_ denotes the error term.

Twelve land cover categories with even a remote likelihood of habitation are included, as shown in Table 1.

**Table 1:** Land cover categories considered potentially habitable and included in the analysis.

Code	Category Name	Description
21	Developed, Open Space	Low-intensity development, mostly vegetation
22	Developed, Low Intensity	Mix of buildings and vegetation (20–49% impervious)
23	Developed, Medium Intensity	Mix of buildings and vegetation (50–79% impervious)
24	Developed, High Intensity	High-density buildings (80–100% impervious)
31	Barren Land	Bare earth, rocks, or sand
41	Deciduous Forest	Trees that shed leaves seasonally
42	Evergreen Forest	Trees that retain leaves year-round
43	Mixed Forest	Mix of deciduous and evergreen trees
52	Shrub/Scrub	Areas dominated by shrubs
71	Grassland/Herbaceous	Dominantly grasses and herbaceous plants
81	Pasture/Hay	Grasses planted for grazing or hay crops
82	Cultivated Crops	Land used for annual/perennial crops

This regression equation includes no intercept, and the coefficients (*b*_*i*_) are constrained to be non-negative (The *nnls* package in R was used to estimate regressions with non-negative coefficients). It is important to note that the purpose of this model is not to achieve statistical significance but to effectively calibrate the population contribution of a pixel for each land cover category within an area.

Since land cover population contributions vary geographically, estimating this model over large areas (e.g., a state or the entire country) would lead to imprecise predictions. To improve accuracy, the model should be estimated for the smallest regions possible. However, as the equation includes 12 explanatory variables, at least 12 block groups are required for estimation. Geographic units such as tracts, ZCTAs, and even counties do not always meet this minimum requirement. To address this, block groups within each state are clustered to maximize the number of clusters while ensuring that each cluster contains at least 12 block groups.

In summary, the clustering process begins with census tracts as the initial clusters, as each tract typically contains 1-9 block groups. Starting with the smallest cluster (i.e., the one with the fewest block groups), clusters are iteratively merged with their neighbors until the required size of at least 12 block groups is reached. Although this algorithm does not guarantee the maximum possible number of clusters meeting the size threshold, its greedy approach (i.e., making locally optimal choices at each step with the aim of finding a global optimum) is fast and effective for this application.

**Fig. 2:**
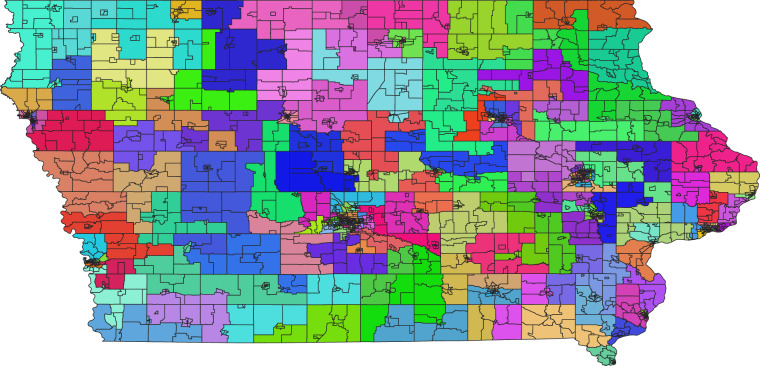
Clusters of Iowa block groups for regionalized land cover regressions, based on 2020 boundaries.

Figure 2 shows the results of the clustering process for Iowa block groups, based on the 2020 census boundaries. The state’s 2,703 block groups are divided into 166 clusters, each represented by a distinct color and containing 12 to 30 contiguous block groups.

The regression equation (Equation (6)) is estimated for each cluster using the block groups it contains. The population for each fraction *F*_*pg*_ within the cluster is then predicted using the resulting coefficients (${\widehat{b}}_{i}$) and the count of land cover pixels in each category within the fraction (*n*_*i*_(*F*_*pg*_)):

7 $$\begin{array}{rcl}\widehat{Pop}({F}_{pg}) & = & {\widehat{b}}_{21}\cdot {n}_{21}({F}_{pg})+{\widehat{b}}_{22}\cdot {n}_{22}({F}_{pg})+{\widehat{b}}_{23}\cdot {n}_{23}({F}_{pg})+{\widehat{b}}_{24}\cdot {n}_{24}({F}_{pg})\\  &  & +{\widehat{b}}_{31}\cdot {n}_{31}({F}_{pg})+{\widehat{b}}_{41}\cdot {n}_{41}({F}_{pg})+{\widehat{b}}_{42}\cdot {n}_{42}({F}_{pg})+{\widehat{b}}_{43}\cdot {n}_{43}({F}_{pg})\\  &  & +{\widehat{b}}_{52}\cdot {n}_{52}({F}_{pg})+{\widehat{b}}_{71}\cdot {n}_{71}({F}_{pg})+{\widehat{b}}_{81}\cdot {n}_{81}({F}_{pg})+{\widehat{b}}_{82}\cdot {n}_{82}({F}_{pg})\end{array}$$

These predictions serve as the weights (*w*_*pg*_) in the population distribution formula in Equation (1):

$${w}_{pg}=\widehat{Pop}({F}_{pg})$$

To reiterate, the RLCR method’s predictions for fraction populations are not used directly. Instead, they function as weights to distribute a block group’s household population among its fractions. This way, the total household population across all fractions within a block group always equals the block group’s household population.

The RLCR method provides reliable population estimates by accounting for regional variations in land cover contributions. As demonstrated in the next section, it significantly outperforms the areal and imperviousness methods, without relying on computationally intensive or black-box machine learning techniques.

## Data Records

The processed datasets (in CSV format) are publicly available on Harvard Dataverse^29^, distributed under the CC0 1.0 Universal license.

These datasets include vote tallies from U.S. general elections, organized by combinations of geographic levels (block groups, census tracts, and ZCTAs), election years (2016 and 2020), and allocation methods (areal, imperviousness, and RLCR).

To avoid data columns empty for most states (i.e., local election candidate vote counts), the data are also organized by state alongside the nationwide datasets.

Each dataset includes an identifier for the census geography (GEOID), county and state FIP codes, population, land area, water area, and the number of contributing precincts (precincts that served as the source of votes for that unit). These are followed by a series of variables in the format GYYEEEPNNN, where G represents a general election, YY denotes the election year (e.g., 16 for 2016, 20 for 2020), EEE specifies the election type (e.g., PRE for presidential, USS for U.S. Senate, and GOV for governor), P indicates the candidate’s party, and NNN consists of the first three letters of the candidate’s last name. If a candidate did not appear on the ballot in a particular census geography, the corresponding value is N/A; otherwise, it reflects the number of votes received by the candidate in that geographic unit.

These datasets can be easily merged with administrative data sources, such as the American Community Survey (ACS), CDC Places, FEMA National Risk Index, and IRS Statistics of Income (SOI), for the corresponding year by using the GEOID variable.

The dataset will be expanded with 2024 election data and future elections as precinct data becomes available.

## Technical Validation

This section presents four types of validation to assess the accuracy and robustness of the proposed vote allocation method. The first subsection evaluates the performance of the underlying population allocation strategies using 2020 census block data. The second compares the constructed vote totals against a ground-truth dataset derived from voter registration files in North Carolina. The third offers a visual assessment of how partisan preferences are preserved during the allocation process. Finally, a practical example illustrates how different methods may yield divergent empirical conclusions.

### Validation of the Population Allocation Method

The vote allocation framework described in the previous section is designed to maintain two internal consistency conditions: (1) the household population assigned to all fractions within a block group must equal the ACS-reported total for that block group, and (2) the total number of votes allocated to all fractions within a precinct must match the precinct’s reported vote total. Thus, as long as the assumption of proportionality between household population and votes within a precinct holds, any inaccuracies stem solely from how household populations are distributed across the precinct-block group intersections.

The key is determining which method (areal, imperviousness, or RLCR) produces the most accurate weights for distributing the population. Benchmarking these methods is challenging because the true population of fractions is unknown. If it were known, there would be no need to estimate weights.

However, fractions are subregions of block groups, as are census blocks (referred to hereafter as just blocks). This makes it possible to compare the weighting methods by testing them on blocks, for which boundaries and population data are available from the 2020 decennial census^23,30^.

For all 8 million-plus blocks in the 2020 U.S. Census, surface areas and habitability values (using equation (5)) were calculated to be used as weights in the areal and imperviousness methods. For the RLCR method, the number of pixels corresponding to different land cover categories within each block was determined, and the regression equation estimated from the parent block group cluster was applied to generate RLCR weights.

Next, for each block, population was distributed from the parent block group using the three weighting methods and compared to the actual population of the block. The following three metrics were calculated (for the MAPE metric, 1 is added to the denominator to avoid division by zero for blocks with a population of zero):

$$\begin{array}{l}\,{\rm{MAE}}=\frac{1}{N}\sum _{b\in {\rm{blocks}}}| {{\rm{Estimated\; Pop}}}_{b}-{{\rm{Actual\; Pop}}}_{b}| \\ \,{\rm{MAPE}}=\frac{1}{N}\sum _{b\in {\rm{blocks}}}\frac{| {{\rm{Estimated\; Pop}}}_{b}-{{\rm{Actual\; Pop}}}_{b}| }{{{\rm{Actual\; Pop}}}_{b}+1}\\ \,{\rm{RMSE}}=\sqrt{\frac{1}{N}\sum _{b\in {\rm{blocks}}}{({{\rm{Estimated\; Pop}}}_{b}-{{\rm{Actual\; Pop}}}_{b})}^{2}}\end{array}$$

Table 2 compares the performance of the three methods across these metrics. The RLCR method consistently outperforms the other two, with lower values for all three error measures. Based on these results, the RLCR dataset is recommended as the primary dataset, while the other two methods can be used for robustness checks.

**Table 2:** Comparison of metrics across three weighting methods for evaluating population distribution accuracy.

	Areal	Imperviousness	RLCR
MAE	28.98	29.37	24.08
RMSE	74.01	76.40	62.23
MAPE	5.25	4.55	4.45

### Allocated Total Votes vs. Ground Truth in North Carolina

To evaluate the accuracy of the vote allocations produced by this paper, a ground truth based on individual-level voter location is necessary. North Carolina is the only state that publicly provides, without fees or formal request procedures, both full voter histories that indicate which elections each registered voter participated in over the past ten years^31^, and voter registration snapshots from the time of each election that include the voter’s residential address^32^. Although these records do not reveal individual vote choices, they enable the construction of total vote counts within census geographies, particularly at the census tract level.

From the voter history file, all individuals (5.52 million) who participated in the “11/03/2020 GENERAL” election were identified and matched to the voter registration snapshot from the same date using a unique voter identifier (NCID). Residential addresses were then geocoded using the Census Bureau’s batch geocoding service (https://geocoding.geo.census.gov/geocoder/locations/addressbatch?form); approximately 3.8% of addresses did not produce a match. For the remaining voters, geographic coordinates were overlaid onto census tract shapefiles^23^ to determine each individual’s corresponding tract. Lastly, voters were aggregated by tract to construct a “near” ground truth, accounting for ungeocoded addresses.

The ground truth vote totals were compared to this paper’s tract-level estimates. Evaluation metrics—mean absolute error (MAE), root mean squared error (RMSE), and mean absolute percentage error (MAPE)—are reported in Table 3.

**Table 3:** Comparison of tract-level total vote estimates against ground truth data derived from individual voter records in North Carolina.

Metric	Areal	Imperviousness	RLCR
MAE	209.00	202.76	185.92
RMSE	286.23	273.95	253.18
MAPE	12.72	12.44	11.59

RLCR achieved the highest accuracy among the three methods, with an MAE of 186 votes per tract (relative to an average of approximately 2,006 total votes per tract). The MAPE was 11.59

While the reported MAPE may appear relatively high, it must be interpreted in the context of several unavoidable sources of error that influence the ground truth itself. First, 3.8% of residential addresses could not be geocoded and were excluded from the analysis. Second, 1.3% of voters were registered in precincts that did not match the precincts containing their geocoded coordinates, indicating possible registration anomalies or boundary mismatches. Third, when using geocoded voter locations to reconstruct precinct-level totals, a 5.3% MAPE was observed when compared to the official precinct vote counts. These compounding discrepancies introduce uncertainty into the ground truth and collectively limit the attainable accuracy of any allocation method. When these factors are taken into account, the performance of the proposed methods remains strong and well within reasonable bounds.

### Visual Validation

**Fig. 3:**
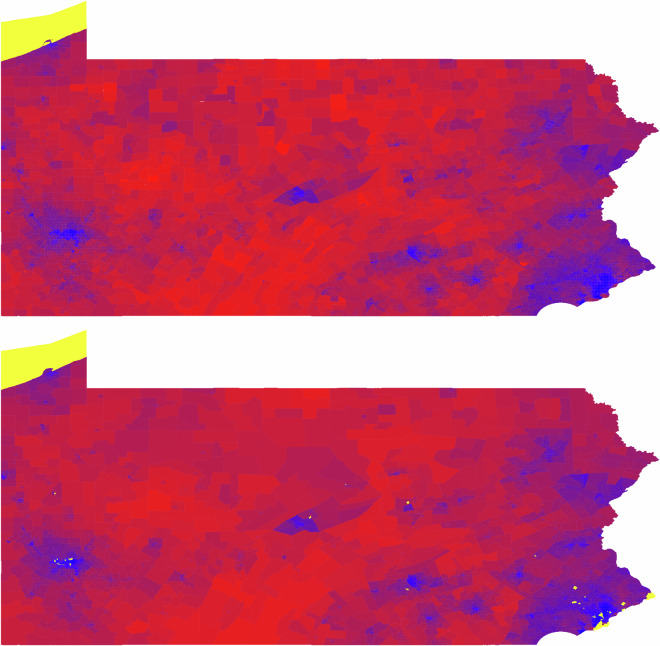
Comparison of 2020 presidential election margins in Pennsylvania at the precinct level (top) and after allocation to census tracts using the RLCR method (bottom). Bluer areas indicate higher Democratic margins and redder areas indicate higher Republican margins. Yellow areas correspond to precincts with zero recorded votes or census tracts with zero household population.

As a visual validation, Fig. 3 compares the 2020 presidential election margins at the precinct level (top) and after allocation to census tracts using the RLCR method (bottom) for Pennsylvania. The close resemblance between the two maps suggests that the allocation process preserves underlying partisan patterns and does not introduce systematic distortion.

### Application Example

One may question whether these three methods produce substantially different outcomes in research. To highlight the importance of using the more precise RLCR allocation technique over its alternatives, a simple voter turnout model for the 2020 presidential election is presented below.

The constructed census tract-level voting data^29^ was merged with the 2020 American Community Survey (ACS) data profiles^14^. Specifically, the Demographic and Housing Estimates (DP05) and Selected Economic Characteristics (DP03) profiles were used. The merge was performed using the GEOID variable, a unique identifier for census tracts. The final merged dataset includes 83,661 tracts across the conterminous United States.

Voter turnout for census tract *i* is modeled as a function of its median age (*age*_*i*_), median household income in thousands of dollars (*income*_*i*_), the percentage of the population with a college degree or higher (*college*_*i*_), and the percentage of the population identified as racial/ethnic minorities (*minority*_*i*_).

$${{\rm{turnout}}}_{i}={\beta }_{0}+{\beta }_{1}\cdot {{\rm{age}}}_{i}+{\beta }_{2}\cdot {{\rm{income}}}_{i}+{\beta }_{3}\cdot {{\rm{college}}}_{i}+{\beta }_{4}\cdot {{\rm{minority}}}_{i}+{\in }_{i}$$

Voter turnout for each tract is defined as the percentage of total votes cast relative to the voting-eligible population. Total votes in the presidential election are calculated as the sum of variables prefixed with G20PRE, which represents presidential election outcomes for all candidates on the ballot, including write-ins where available. The voting-eligible population (American citizens aged 18 and older) is explicitly provided in the ACS.

The voter turnout variables constructed using vote counts from the areal, imperviousness, and RLCR methods are named turnout_1_, turnout_2_, and turnout_3_, respectively.

Tracts missing any of the four explanatory variables or with a voting population of fewer than 50 individuals were excluded from the analysis, reducing the number of observations by 1,528.

As previously mentioned, the analysis is also conducted separately for rural and metropolitan subsets of the data. To define these subsets, population density for each tract is calculated as the total population divided by the land (non-water) area. The density variable is then divided into quintiles. Tracts in the lowest quintile are classified as rural, while those in the highest quintile are classified as metropolitan.

Table 4 presents the t-statistics (calculated as the coefficient estimate divided by its standard error) along with the percentage differences in t-values between methods 1 (areal) and 3 (RLCR), and methods 2 (imperviousness) and 3, for each variable. The results show that t-statistics can differ by as much as 55% between methods 1 and 3, while the difference between methods 2 and 3 is smaller (up to 6%).

**Table 4:** T-statistics and percentage differences between methods for voter turnout determinants.

Subset	Variable		turnout_1_	turnout_2_	turnout_3_	Diff 1 & 3	Diff 2 & 3
All	age	t-stat	55.84	65.33	65.79	15.13%	0.7%
	income	t-stat	71.76	81.28	81.75	12.22%	0.58%
	college	t-stat	52.17	47.36	50.00	4.35%	5.28%
	minority	t-stat	− 39.29	− 41.21	− 41.71	5.79%	1.2%
Rural	age	t-stat	15.93	20.46	19.60	18.7%	4.39%
	income	t-stat	8.47	19.07	18.69	54.66%	2.06%
	college	t-stat	28.23	28.96	30.12	6.27%	3.84%
	minority	t-stat	− 13.56	− 12.82	− 12.97	4.53%	1.15%
Metro	age	t-stat	23.20	23.12	24.02	3.43%	3.74%
	income	t-stat	32.18	31.17	31.71	1.49%	1.72%
	college	t-stat	19.04	19.04	19.98	4.72%	4.71%
	minority	t-stat	− 5.41	− 4.84	− 4.59	17.9%	5.61%

The discrepancies in t-statistics between methods could alter statistical significance in studies, particularly in rural areas. These differences are more pronounced in rural areas, where tracts are larger and population distributions are more uneven. Therefore, academic studies of electoral behavior can benefit from the improved precision provided by the RLCR.

## Data Availability

The custom code to replicate these results is available on GitHub (https://github.com/fekrazad/precinct-to-census-mapping). The GIS operations (including the intersection of precinct and census geography boundaries, area calculations, and overlaying with land cover and imperviousness rasters) were automated using PyQGIS. The GIS outputs were processed alongside population and voting data in R, using the Tidyverse and DataTable packages to generate the final tabular datasets. This methodology is replicable using modest computational resources and free tools, including R, Python, and QGIS.

## References

[1] Kahane, L. H. Determinants of county-level voting patterns in the 2012 and 2016 presidential elections. *Applied Economics* **52**(33), 3574–3587 (2020).

[2] Parzuchowski, A. S., Peters, A. T., Johnson-Sasso, C. P., Rydland, K. J. & Feinglass, J. M. County-level association of covid-19 mortality with 2020 united states presidential voting. *Public Health* **198**, 114–117 (2021). 34416573 10.1016/j.puhe.2021.06.011 PMC9451615

[3] Barnett, M. L., Gaye, M., Jena, A. B. & Mehrotra, A. Association of county-level prescriptions for hydroxychloroquine and ivermectin with county-level political voting patterns in the 2020 us presidential election. *JAMA Internal Medicine* **182**(4), 452–454 (2022). 35179552 10.1001/jamainternmed.2022.0200 PMC8980920

[4] Kinsella, C., McTague, C. & Raleigh, K. Closely and deeply divided: Purple counties in the 2016 presidential election. *Applied Geography* **127**, 102386 (2021).

[5] Pritchard, Z. D. & Mills, S. Renewable energy requirements on the ballot: An analysis of county-level voting results. *Energy Policy* **148**, 111949 (2021).

[6] Curtis, L. H., Hoffman, M. N., Califf, R. M. & Hammill, B. G. Life expectancy and voting patterns in the 2020 u.s. presidential election. *SSM - Population Health* **15**, 100840 (2021). 34169139 10.1016/j.ssmph.2021.100840 PMC8209240

[7] Ang, D. Do 40-year-old facts still matter? long-run effects of federal oversight under the voting rights act. *American Economic Journal: Applied Economics* **11**(3), 1–53 (2019).

[8] Alexander, S. *et al*. The impact of extreme precipitation events and their variability on climate change beliefs in the american public. *Weather, Climate, and Society* **15**(4), 863 – 879 (2023).

[9] Layman, G. C., Allen, L. G., R. G. Kirk, J., Z. C. Marsh, W. & Radcliff, B. The pandemic and political behavior: Staying the course. *PS: Political Science & Politics* **57**(3), 414–419 (2024).

[10] Zandbergen, P. A. & Ignizio, D. A. Comparison of dasymetric mapping techniques for small-area population estimates. *Cartography and Geographic Information Science* **37**(3), 199–214 (2010).

[11] Leyk, S., Buttenfield, B. P., Nagle, N. N. & Stum, A. K. Establishing relationships between parcel data and land cover for demographic small area estimation. *Cartography and Geographic Information Science* **40**(4), 305–315 (2013).

[12] Baynes, J., Neale, A. & Hultgren, T. Improving intelligent dasymetric mapping population density estimates at 30 m resolution for the conterminous united states by excluding uninhabited areas. *Earth System Science Data* **14**(6), 2833–2849 (2022). 36213148 10.5194/essd-14-2833-2022 PMC9534036

[13] Swanwick, R. H. *et al*. Dasymetric population mapping based on us census data and 30-m gridded estimates of impervious surface. *Scientific Data* **9**(1), 523 (2022). 36030258 10.1038/s41597-022-01603-z PMC9422266

[14] U.S. Census Bureau. American community survey 5-year estimates, data profiles. https://data.census.gov/table?y=2020&d=ACS+5-Year+Estimates+Data+Profiles (2020).

[15] Internal Revenue Service. SOI Tax Stats - Individual Income Tax Statistics - ZIP Code Data. https://www.irs.gov/statistics/soi-tax-stats-individual-income-tax-statistics-zip-code-data-soi Accessed: 2025-04-17 (2025).

[16] Centers for Disease Control and Prevention. PLACES: Local Data for Better Health - Place Data 2024 release. https://data.cdc.gov/500-Cities-Places/PLACES-Local-Data-for-Better-Health-Place-Data-202/eav7-hnsx Accessed: 2025-04-17 (2024).

[17] Federal Emergency Management Agency. National Risk Index Data Resources. https://hazards.fema.gov/nri/data-resources Accessed: 2025-04-17 (2023)

[18] Voting and Election Science Team (VEST). 2016 precinct-level election results. 10.7910/DVN/NH5S2I, 2018. Version V94.

[19] MIT Election Data and Science Lab (MEDSL). Precinct-level returns 2020 by individual state. 10.7910/DVN/NT66Z3 Version V6, UNF:6:aViWPnsxmDD+s1GuFrrdpA== (2022).

[20] Amos, B., Gerontakis, S. & McDonald, M. United states precinct boundaries and statewide partisan election results. *Scientific Data* **11**(1), 1173 (2024). 39472432 10.1038/s41597-024-04024-2 PMC11522301

[21] Baltz, S. *et al*. American election results at the precinct level. *Scientific Data* **9**(1), 651 (2022). 36329037 10.1038/s41597-022-01745-0 PMC9633829

[22] U.S. Census Bureau. 2016 tiger/line shapefiles. https://www.census.gov/geographies/mapping-files/time-series/geo/tiger-line-file.2016.html (2016).

[23] U.S. Census Bureau. 2020 tiger/line shapefiles. https://www.census.gov/geographies/mapping-files/time-series/geo/tiger-line-file.2020.html (2020).

[24] U.S. Census Bureau. American community survey 5-year estimates, detailed tables. https://data.census.gov/table?y=2016&d=ACS+5-Year+Estimates+Detailed+Tables (2016).

[25] U.S. Census Bureau. American community survey 5-year estimates, detailed tables. https://data.census.gov/table?y=2020&d=ACS+5-Year+Estimates+Detailed+Tables (2020).

[26] U.S. Geological Survey. Annual national land cover database (nlcd) collection 1: Land cover. 10.5066/P94UXNTS (2024).

[27] U.S. Geological Survey. Annual national land cover database (nlcd) collection 1: Fractional impervious surface. 10.5066/P94UXNTS (2024).

[28] U.S. Geological Survey. Annual national land cover database (nlcd) collection 1: Impervious descriptor. 10.5066/P94UXNTS (2024).

[29] A., Fekrazad Reallocating u.s. election results from precincts to census geographies. 10.7910/DVN/Z8TSH3 Version V2 (2025).

[30] U.S. Census Bureau. 2020 decennial census redistricting data (public law 94-171). https://data.census.gov/table/DECENNIALPL2020 (2021).

[31] North Carolina State Board of Elections. Voter history data. https://www.ncsbe.gov/results-data/voter-history-data Accessed: 2025-04-15 (2025).

[32] North Carolina State Board of Elections. Voter registration snapshots. https://dl.ncsbe.gov/index.html?prefix=data/Snapshots/ Accessed: 2025-04-15 (2025).

